# Pancreatic Duct Variations and the Risk of Post-Endoscopic Retrograde Cholangiopancreatography Pancreatitis

**DOI:** 10.7759/cureus.10445

**Published:** 2020-09-14

**Authors:** Ademola S Ojo

**Affiliations:** 1 Department of Anatomical Sciences, St. George's University School of Medicine, St. George's, GRD

**Keywords:** pancreatic duct, pancreatitis, endoscopic retrograde cholangiopancreatography, complications

## Abstract

Endoscopic retrograde cholangiopancreatography (ERCP) is an important diagnostic and therapeutic procedure in the management of biliary and pancreatic disorders. Despite advances in ERCP facilities and techniques, pancreatitis remains the most common and feared complication of this procedure. The technical challenges of ERCP could be further compounded by variations in the configuration of the pancreatic ductal system. As a result, the knowledge of these variations and their potential role in the development of post-ERCP pancreatitis (PEP) is essential to any successful risk reduction strategy. This review provides an overview of the anatomy and embryological basis of pancreatic duct variations, as well as explore the different types and prevalence of these variations. Also, we discuss the mechanisms of PEP and provide evidence supporting a link between the variations and PEP using published data

## Introduction and background

Since 1968 when William McCune, an obstetrician performed the first procedure, endoscopic retrograde cholangiopancreatography (ERCP) has become an important diagnostic as well as a therapeutic procedure in the management of biliary and pancreatic disorders [1]. Although less commonly performed when compared to esophagogastroduodenoscopy and colonoscopy, an estimated 350,000 to 500,000 ERCPs are performed in the United States annually [1,2]. Choledocholithiasis, obstructive cholangiocarcinoma, obstructive jaundice, biliary pancreatitis, and biliary stricture are common indications for this procedure [3,4]. Other conditions such as pancreatic or biliary duct leaks, chronic pancreatitis, pancreatic stones as well as pancreatic strictures are less common indications [3,4]. Due to the technical difficulties associated with this procedure, the rate of complications from ERCP is higher when compared to other traditional gastrointestinal endoscopies. Common complications of ERCP include pancreatitis, perforations, post-sphincterotomy bleeding, biliary/pancreatic ductal injuries, cholangitis, cholecystitis as well as cardiopulmonary complications related to anesthesia [5].

Post-ERCP pancreatitis (PEP) is the most common of these complications occurring in 2% to 9% of ERCP, rising to 30% in patients undergoing high-risk procedures [5,6]. Although variations occur in the diagnostic criteria used across different regions and institutions, the most widely accepted consensus criteria define PEP as (1) the presence of abdominal pain suggestive of pancreatitis, (2) a laboratory finding of elevated serum amylase at least three times the upper limit of normal 24 hours post-ERCP, and (3) which requires an additional or unplanned hospitalization for two or more days [7]. Many predisposing factors for PEP have been identified. These include patient factors, the nature of the procedure, and operator-related factors [6]. While much focus has been on patient factors such as the age, gender, and sphincter of Oddi dysfunction in the occurrence of PEP, the impact of pancreatic duct variations on the risk of developing PEP has remained a subject of debate. Pancreatic duct variations are relatively common, occurring in 5.7% of the population [8]. Many of these variations have been associated with pathologies such as acute pancreatitis, chronic pancreatitis, and pancreatic tumors [9,10]. This review provides an overview of the anatomy and embryological basis of pancreatic duct variations, as well as explore the different types and prevalence of these variations. Also, we discuss the mechanisms of PEP and provide evidence for a possible link between the variations and PEP using published data.

## Review

Anatomy and embryology of the pancreatic ducts

**Figure 1:**
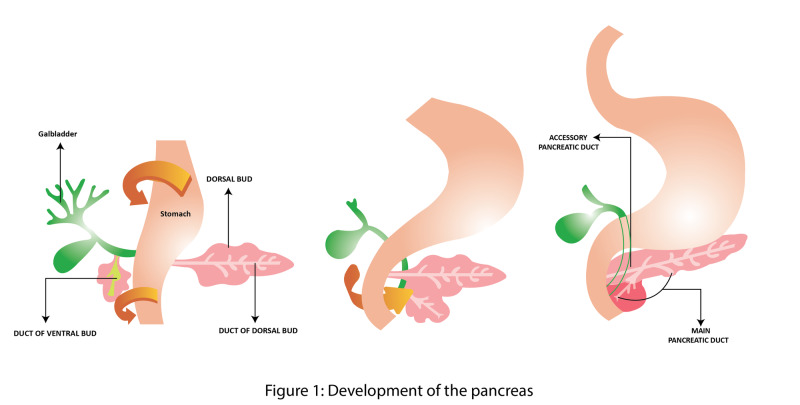
Development of the pancreas

The pancreas develops as an endodermal outgrowth from two pancreatic buds which develop at the junction of the foregut and midgut around the second and third weeks of intrauterine life (Figure 1) [11]. The smaller ventral bud has two lobes, right and left, of which the left lobe regresses, while the right lobe persists as the ventral bud which grows into the ventral mesentery [12]. The larger dorsal bud grows posteriorly into the dorsal mesentery [11]. The rotation of the foregut (stomach and duodenum) ensures the duodenum becomes a retroperitoneal structure, carrying with it the developing pancreas. It also results in a dorsal migration of the ventral bud from the right to the left side of the embryo. The two pancreatic buds fuse to form a single adult pancreas at the 7th week of intrauterine life with the ventral bud contributing the uncinated process and part of the head, while the tail, body, neck, and the rest of the head originate from the dorsal bud [13]. Similarly, the duct of the ventral bud and distal part of the duct of the dorsal bud anastomose to form the main pancreatic duct (of Wirsung). The duodenal end of the dorsal bud duct degenerates but oftentimes persist as the accessory pancreatic duct (of Santorini) in up to 95% of the population [14].

 

Anatomically, the pancreas is a retroperitoneal organ made up of exocrine and endocrine secretory components. It has a head which lies in the concavity of the duodenum, a neck which lies anterior to the commencement of the portal vein, a body which extends to the hilum of the left kidney, and a tail which lies in the splenorenal ligament, extending to the splenic hilum [11]. The main pancreatic duct extends from the tail to the head of the gland, draining most of the gland and joining the bile duct at the hepatopancreatic ampulla to drain into the second part of the duodenum. This ampulla, as well as the end of the two ducts, are surrounded by the ampullary sphincter (of Oddi). The ampulla opens into the second part of the duodenum at the major duodenal papilla. The accessory pancreatic duct when present drains the uncinated process and part of the head. It opens into the duodenum at the minor duodenal papillae in up to 90% of cases, proximal to the major papillae, or drain directly into the main duct [14].

Pancreatic duct variations: classification and prevalence

Anatomical variations of the pancreatic duct are common, many of which are detected incidentally on imaging studies for other conditions [15]. Computed tomography (CT) scan, magnetic resonance cholangiopancreatography (MRCP), and ERCP are common imaging modalities used in assessing the pancreas. MRCP and ERCP are more accurate than CT scans in pancreatic duct imaging, with the latter being more reliable in visualizing pancreatic pathologies such as pancreatitis and tumors [15]. Variations could occur in configuration, course, number, and termination into the duodenum.

Pancreatic Duct Configuration Variations

**Figure 2:**
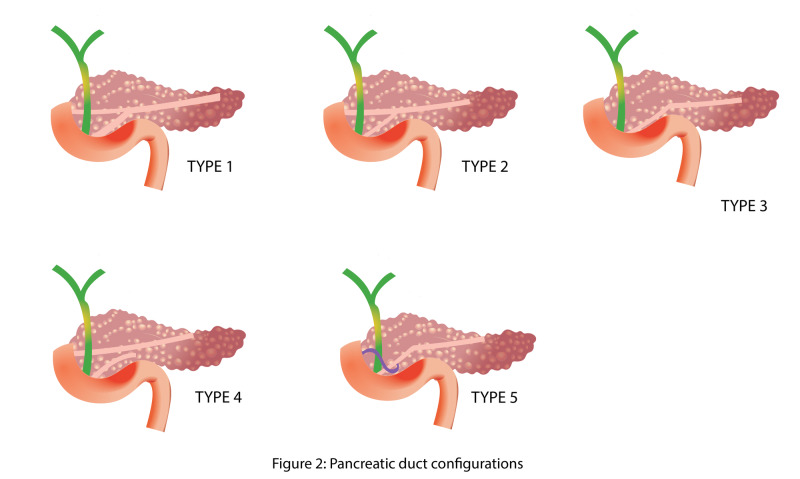
Pancreatic duct configurations

A widely accepted classification system for pancreatic ductal configuration recognizes five classes of ductal configuration (Figure 2) [8].

Type 1: A bifid configuration with the main pancreatic duct (MPD) draining into the duodenum at the major papilla, communicating with a well-defined accessory pancreatic duct (APD), which drains into the duodenum at the minor papilla.

Type 2: A bifid configuration with the APD as the dominant drainage. APD and MPD drains into the minor and major papilla, respectively.

Type 3: Absent or rudimentary APD.

Type 4: A pancreas divisum, characterized by non-fusion of the ventral and dorsal pancreatic ducts with the MPD draining into the minor papilla and APD into major papilla. There are three subtypes: (a) absent communication between the ventral and dorsal duct, (b) no ventral duct and (c) small communication between the ducts.

Type 5: Ansa pancreatica in which the proximal APD at its junction with MPD is obliterated, and replaced by an S-shaped duct arising from the MPD which terminate in or around the minor papilla.

Types 1, 2 and 3 are regarded as 'normal' anatomy [15]. A recent systematic review that analyzed the pancreatic duct anatomy of 8,260 individuals found normal anatomy (types 1-3) in 94.3% of the cases, pancreas divisum (type 4) in 4.5%, and ansa pancreatica (type 5) in 0.25% of individuals [8].

Annular Pancreas, Duplications and Cystic Dilatation of the Pancreatic Duct

Annular pancreas is a congenital anomaly of the pancreas in which a ring of pancreatic tissue partially or completely surrounds the duodenum. An abnormal fusion of the tip of the ventral pancreatic bud to the duodenum and the subsequent malrotation of the ventral bud has been postulated as the developmental anomaly underlying the annular pancreas [16]. The estimated incidence is 1 in 1000 to 1 in 6000 people [17]. ERCP studies show a pancreas divisum is the most common aberrant ductal pattern in annular pancreas, occurring in 29%-46% of cases [16,17]. This is higher than the incidence of pancreas divisum in the general population. Duplication anomalies of pancreatic ducts are less common compare to abnormalities of pancreatic bud fusion such as pancreas divisum. Previous ERCP studies reported duplication abnormalities in 4.5% to 5.5% of individuals with partial duplications occurring more often than complete variations [9,18]. Duplication anomalies are usually associated with loop, ring, N-shaped, and spiral abnormalities of the pancreatic duct [18]. Cystic dilatation of the pancreatic duct is a rare abnormality with an unknown incidence [19]. Cystic dilation at the termination of the dorsal duct referred to as 'santorinicele' is associated with pancreas divisum and has been thought to occur due to the obstruction to the flow of pancreatic secretion and weakness of the duodenum at the point of termination of the duct [20].

Aberrant Pancreaticobiliary Ductal Junction Formation

The pancreatic duct and common bile duct drain into the duodenum via a common channel (in 55% to 82% of individuals) or separately [21]. When drainage occurs through a common channel, union of the pancreatic and common bile ducts occurs within the duodenal wall at the major papilla, with the sphincter of Oddi located at the distant end of the ducts where it regulates pancreatic and biliary secretion [22]. Aberrant pancreaticobiliary ductal junction formation ensues if the union of the two ducts occurs outside the wall of the duodenum resulting in a long common channel (>15mm) [21]. This abnormality has been reported in up to 2.6% of individuals undergoing ERCP [23]. It is associated with the reflux of bile into the pancreatic duct, as well as pancreatic juice reflux into the bile duct [24]. Congenital choledochal cyst and gallbladder malignancy are common findings in individuals with this anomaly [22].

Post-ERCP pancreatitis: pathophysiology and features

Pancreatitis following ERCP occurs through a complex interaction of many factors. The combination of predisposing factors and a suitable trigger event result in pancreatic inflammation similar to pancreatitis from other etiologies.

Predisposing Factors for Post-ERCP Pancreatitis

The risk of PEP can be due to patient-, procedure- or physician-related factors [6]. Patient-related factors include female gender, younger age (<60 years), and sphincter of Oddi dysfunction (SOD) [25,26]. SOD is characterized by recurrent abdominal pain due to structural or functional abnormality of the sphincter of Oddi in an individual without any obvious biliary or pancreatic pathology [6]. It occurs more often in women, and it independently triples the risk of PEP [6]. Also, a prior episode(s) of acute pancreatitis or PEP independently increases the risk of PEP [27]. The nature of the procedure is equally an important predictor of the risk of PEP. Contrast injection into the pancreatic duct increases the risk of PEP, with an incremental rise in risk with repeated injections [5]. In addition, papillary trauma, often resulting from difficult cannulation raises the risk of PEP, independent of the number of times contrast was injected into the pancreatic duct [28]. Also, the risk of PEP increases cumulatively as the number of attempts at cannulation increases [28]. Pancreatic sphincterotomy which facilitates access into the pancreatic duct, as well as balloon dilation of the biliary sphincter, has been shown to increase the risk of PEP [26,29]. Cannulation and contrast injection into the minor papilla has also been reported to increase the risk of PEP [27,30]. Thermal injury from electrosurgical devices contributes to the risk of PEP following pancreatic sphincterotomy [6]. Besides, the case volume and experience of the endoscopist have been considered in assessing the risk of PEP. While many studies have shown an increased risk of complications from ERCP in low-volume centers, the relationship between case volume and risk of PEP has not been fully established [28,31]. However, trainee involvement in ERCP has been reported as an independent risk factor for PEP in a study, highlighting the role of expertise in the prevention of PEP [25].

Inciting Events for Post-ERCP Pancreatitis

Although the exact trigger that sets off the chain of events leading to PEP is not clear, many mechanisms have been proposed. Pancreatic duct blockage has been considered a prominent event in the development of PEP [5]. This could result from post-cannulation papillary edema and/or sphincter of Oddi spasm [32]. Pancreatic duct blockage prevents the outflow of pancreatic juice resulting in increased intrapancreatic duct pressure as well as accumulation of pancreatic juice within the gland interstitium. This leads to premature activation of pancreatic enzymes within the gland. Pancreatic duct injury resulting from thermal injury, mechanical trauma due to guidewire manipulation, as well as chemical, allergic or hydrostatic injury from contrast injection, have been considered as inciting events for PEP, either alone or in combination with other factors [6]. The possible role of secondary bacteria colonization and infection of the pancreatic duct from instrumentation have also been considered, with this hypothesis supported by the report of a lower risk of PEP in patients who received prophylactic antibiotics in a randomized controlled trial [33].

Mechanism and Features of Pancreatic Inflammation

Regardless of the inciting event, pancreatic inflammation results from premature activation of pancreatic enzymes within the pancreas [32]. Pancreatic digestive enzymes are synthesized and released in secretory granules as proenzymes (zymogens), which are activated in the lumen of the small intestine by trypsin, a pancreatic enzyme formed from trypsinogen through the action of duodenal enteropepsidase (enterokinase). Intrapancreatic activation of trypsin leads to the activation of other pancreatic proenzymes such as chymotrypsinogen, proelastase, procarboxypepsidase, and prophospholipase. The combined effect of these digestive enzymes results in the degradation of fat cells, elastic fibers in the wall of blood vessels, as well as the activation of prekallikrein; a serine protease capable of activating the clotting cascade and complement system [34]. The overall effect is pancreatic inflammation with proteolysis, small-vessel thrombosis, fat cell degradation, and pancreatic acinar destruction [34]. The inflammatory process could be limited to the pancreas, extend to peripancreatic tissues, or result in a systemic inflammatory response [6].

Pancreatitis occurring following ERCP has similar features with pancreatitis from other causes [5]. Persistent abdominal pain of sudden onset is a key manifestation, often radiating to the back. The severity ranges from mild discomfort to severe and incapacitating. This is often associated with anorexia, nausea, or vomiting. Increased serum lipase and/or amylase at least three times the upper limit of normal are important laboratory findings in acute pancreatitis [34]. However, the level of these enzymes does not correlate with disease severity [35]. Contrast-enhanced computed tomography scan is the imaging modality of choice, often revealing features of pancreatic inflammation and necrosis, although normal imaging can be found in mild disease. Elevated enzymes without abdominal pain as well as abdominal pain in the absence of elevated enzymes could occur following ERCP, none of which represent clinical pancreatitis [6].

Post-ERCP pancreatitis in aberrant pancreatic duct anatomy

Pancreas divisum, ansa pancreatica, and aberrant pancreaticobiliary junction constitute the most commonly encountered abnormal variations of the pancreatic ductal system. While the role of other patient factors in the development of PEP has been extensively studied, few studies have sought to establish a correlation between these variations and the risk of PEP.

Pancreas Divisum and PEP

Pancreas divisum is one of the most frequently encountered variations of the pancreatic duct and has been considered in evaluating the risk of PEP by many studies with contrasting results. A prospective multicenter study by Freeman et al. evaluated 1963 cases of ERCP and found 58 patients (3%) have a pancreas divisum [26]. In that study, univariate analysis shows the risk of PEP significantly increases in pancreas divisum (P=0.001). However, this was not supported by multivariate analysis. This finding is similar to the report of a multicenter prospective study that analyzed 1115 ERCPs, and found PEP in 15.1% of the cases [25]. In the study, univariate analysis shows an increased risk of pancreatitis in individuals with a pancreas divisum (P=0.002) which was not supported by multivariable analysis. In another study by Jeurnink et al., a multivariable analysis of predictors of PEP demonstrated a significantly increased risk of PEP in pancreas divisum (OR-10.5; CI 1.0-112.8) [36]. An additional systematic review of existing literature by the authors shows a pancreas divisum increases the risk of PEP in a multivariable analysis (pooled OR- 2.2; CI 1.4-3.4). Similarly, Rabenstein et al examine the risk factors for developing complications following endoscopic sphincterotomy in 438 ERCP and reported complications in 7.5% of cases, with pancreatitis the most frequent complication occurring in 4.3% [37]. In the study, multivariate analysis shows a pancreas divisum increases the risk of complications following endoscopic sphincterotomy (P=0.05). Whether the observation from these studies is largely due to the technical challenges associated with this variation is not clear, however, minor papilla cannulation and pancreatic sphincterotomy are often performed in a pancreas divisum, both of which independently increase the risk factors of PEP [6]. Also, the risk of acute pancreatitis in the general population is reported to be higher in individuals with pancreas divisum, further highlighting the complex relationship between PEP and pancreatic divisum [10].

In contrast to the findings of the previously mentioned studies which were performed mostly in adult populations, Deng et al retrospectively reviewed 92 ERCPs performed in pediatric patients and reported pancreas divisum in 49.4% of the cases [38]. PEP was found in 20.7% of all the ERCPs. Univariate analysis of factors associated with PEP development shows no significant association between pancreas divisum and the risk of PEP (P=0.21). However, it is not clear if this finding is due to the age group of the study population or the effect of a smaller sample size.

Ansa Pancreatica and PEP

Since it was first reported by Dawson and Langman in 1961, there have been case reports and series on ansa pancreatica [39-41]. Many studies have hypothesized ansa pancreatica as a risk factor for acute pancreatitis [40,41]. This hypothesis is supported by the findings of a recent case-control study which reported a significantly higher risk of recurrent acute pancreatitis in individuals with ansa pancreatica [39]. However, no correlation was found with a single episode of acute pancreatitis. Unlike pancreas divisum which has been vastly studied and explored, there is limited information on the role of ansa pancreatica in pancreatic pathologies and no study has explored the relationship of this aberrant anatomy and the risk of PEP.

Pancreaticobiliary Ductal Junction Anomaly and PEP

The formation of the pancreaticobiliary junction (PBJ) outside of the duodenal wall has been recognized as a predisposing factor for acute pancreatitis in addition to the risk of a choledochocele due to biliopancreatic and pancreaticobiliary reflux [21]. Reflux of bile into the pancreatic duct is thought to result in premature activation of the pancreatic enzyme leading to pancreatic autodigestion and inflammation [42]. Anomalous PBJ has been linked with an increased risk of recurrent pancreatitis [43]. However, there is limited information on the relationship between this variation and the risk of PEP. Alkhatib et al. retrospectively reviewed 2,218 ERCPs performed on 1050 patients with no prior history of pancreatitis and found anomalous PBJ in 1.1% of the patients [42]. None of the patients developed PEP. The authors hypothesized the absence of PEP in these patients may be due to the recurrent pancreatic ductal injection from biliopancreatic reflux, 'desensitizing' the pancreatic duct to contrast injection-associated pancreatic duct injuries, an important predisposing factor for PEP.

## Conclusions

ERCP is an important diagnostic and therapeutic procedure in the management of biliary and pancreatic disorders. Pancreatitis remains the most common and feared complication of this procedure. The technical challenges of ERCP could be further compounded by variations in the configuration of the pancreatic ductal system. As a result, the knowledge of these variations and their potential role in the development of PEP is essential to any successful risk reduction strategy. There is, therefore, a need for additional studies to further understand the complex relationship between these variations and the risk of PEP. This will help to achieve a more objective risk stratification in patients undergoing ERCP.
